# Air pollution, emerging chemical exposures, and systemic lupus erythematosus: a meta-epidemiology study

**DOI:** 10.3389/fimmu.2025.1613441

**Published:** 2025-10-17

**Authors:** Yaling Xu, Hejing Pan, Wu Chen, Yehang Wang, Xuanlin Li, Qiaoding Dai, Lin Huang

**Affiliations:** ^1^ College of Basic Medical Science, Zhejiang Chinese Medical University, Hangzhou, China; ^2^ the First Affiliated Hospital of Zhejiang Chinese Medical University, Hangzhou, China; ^3^ Zhejiang Provincial Hospital of Chinese Medicine, Hangzhou, China

**Keywords:** air pollution, systemic lupus erythematosus, environmental exposure, particulate matter, meta-epidemiology study

## Abstract

**Objectives:**

This meta-analysis evaluated the direction and strength of associations between air pollution, emerging chemical pollutants, and systemic lupus erythematosus (SLE) incidence, clarifying distinct relationships by pollutant type.

**Method:**

By utilizing medical subject headings and keywords from the PubMed and EMBASE databases, a thorough search was conducted for published observational studies linking air pollution and SLE from inception until August 2024. The Newcastle-Ottawa Scale (NOS) was utilized to evaluate the quality of the studies. Statistical analyses were performed using STATA software (version 14.0), with the assessment of publication bias conducted through funnel plots and Egger’s test.

**Result:**

This meta-analysis encompassed 8 studies published between 2018 and 2024, involving a total of 1,390,348 individuals. We assessed exposure to standard air pollutants and emerging chemical pollutants, specifically including perfluoroalkyl and polyfluoroalkyl substances (PFASs, a type of persistent chemical widely used in nonstick cookware and waterproof products) and bisphenol compounds (BPs, a synthetic chemical primarily used in plastic products and resins). These eight studies identified significant positive associations between SLE incidence and exposure to PM_2.5_ [OR = 1.16, 95% CI (1.02-1.32), I^2^ = 62.4%, p=0.031], NO_2_ [OR = 1.24, 95% CI (1.11-1.38), I² = 0.0%, P = 0.603], and PFASs [OR = 2.47, 95% CI (1.54-2.57)], while O_3_ exhibited a negative association [OR = 0.83, 95% CI (0.70-0.98), I² = 19.3%, P = 0.290]. No significant links were found for PM_10_ [OR = 1.11, 95% CI (0.90–1.36), I² = 66.3%, P = 0.031], SO_2_ [OR = 0.99, 95% CI (0.66-1.48), I² = 79.0%, P = 0.001], and BPs [OR = 1.26, 95% CI (0.80-1.99)]. Sensitivity analyses supported robustness without evidence of publication bias.

**Conclusion:**

The results of this meta-analysis suggest that air pollutants PM_2.5_ and NO_2_ may be potential environmental risk factors for SLE, while the negative correlation with O_3_ requires further research to validate its potential mechanisms. It is worth noting that although a study on PFASs showed a strong association with SLE, this finding requires further evidence due to the limited number of relevant studies currently available. These findings imply that improving air quality and strengthening regulation of emerging pollutants may reduce the disease burden of SLE. Based on the current strength of evidence, public health policies should prioritize reducing population exposure levels to PM_2.5_ and NO_2_, which may help reduce the potential risk of SLE onset. Concurrently, larger-scale studies should be conducted to confirm the association between other environmental pollutants such as PFASs and SLE, providing more comprehensive scientific evidence for the development of targeted environmental health policies.

**Systematic Review Registration:**

https://www.crd.york.ac.uk/PROSPERO/, PROSPERO (CRD42024581931).

## 1 Introduction

Systemic lupus erythematosus (SLE) is a chronic autoimmune disease. It features autoantibodies, immune complexes, and activated autoreactive B and T lymphocytes (1). Globally, SLE is estimated to have an incidence of 5.14 cases per 100,000 person-years, with approximately 0.40 million new cases diagnosed annually (2). SLE can lead to multi-organ damage, presenting symptoms ranging from skin lesions and joint issues to severe fatigue, cognitive impairment, renal disease, and thrombosis (3). SLE often causes diverse joint problems, affecting up to 95% of patients (4). These chronic joint issues in SLE patients reduce quality of life, impact functional performance, and are among the most disabling aspects of SLE (5). These issues significantly reduce quality of life and functional capacity, with 12% developing permanent joint damage and 19%-40% experiencing work disability within 5 years of onset (6). The complex etiology of SLE involves genetic, infectious, lifestyle, and environmental factors, with environmental pollution being a significant risk factor impacting disease onset and progression (7, 8).

Global industrialization has made air pollution a major health concern (9). Airborne pollutants such as particulate matter, sulfur dioxide, and nitrogen oxides—primarily affect respiratory health and induce oxidative stress and inflammatory responses (10). Current evidence on air pollution and SLE reveals significant variations by pollutant type. Particulate matter (PM_2.5_ and PM_10_) demonstrates more consistent associations with SLE outcomes—ranging from disease activity (11) to serological autoantibody levels (12). In contrast, gaseous pollutants (NO_2_, SO_2_, O_3_) exhibit weaker or null associations in multiple studies (13, 14), and the findings from these studies are less conclusive. In addition to air pollutants, increasing evidence suggests that other environmental chemicals may also be involved in the pathogenesis of SLE. Bisphenol compounds (e.g., BPA, BPS), widely used in plastics and consumer products, may disrupt immune homeostasis through estrogen-like activity and epigenetic regulation (15, 16). Similarly, perfluoroalkyl and polyfluoroalkyl substances (PFASs)—persistent chemicals found in waterproof coatings and drinking water—have been linked to immune dysfunction and autoantibody production (17). Although epidemiological data on these chemicals remain limited, their widespread environmental presence and biological persistence warrant further investigation alongside traditional air pollutants.

While previous studies have mainly concentrated on occupational hazards, smoking, and alcohol consumption as non-shared factors, recent research indicates that environmental factors contribute significantly to the phenotypic variance observed in SLE. Shared environmental factors account for 25.8% of this variance, and non - shared ones for 30.3% (18), highlighting the need to explore non-occupational exposures like air pollution (19).

While previous meta-analyses have explored the association between air pollution and autoimmune diseases, there have been few studies specifically targeting SLE for a comprehensive assessment covering multiple pollutants, including emerging chemical exposures. Existing studies, such as the work by Rezayat et al. (20), have primarily emphasized the association between PM_2.5_ and SLE disease activity, but have lacked systematic analysis of other key pollutants, such as NO_2_, PFASs, and BPs. This study conducted the first meta-epidemiological analysis to quantify the direction and strength of associations between multiple air pollutants and SLE incidence, as well as to explore differential effects across population subgroups and study designs. By integrating updated epidemiological data and expanding the scope of the study to include emerging chemicals, our findings help clarify the potential associations between SLE and environmental factors, which may inform public health strategy and promote further research into potential mechanisms.

## 2 Methods

This meta-analysis was conducted in accordance with the Preferred Reporting Items for Systematic Reviews and Meta-Analyses (PRISMA) guidelines. The protocol was pre-registered in the International Register of Prospective Systematic Reviews (PROSPERO) under approval number CRD42024581931.

### 2.1 Data sources

We systematically searched the PubMed and EMBASE databases using Medical Subject Headings (MeSH for PubMed and Emtree for EMBASE) and relevant keywords with the following strategy before August 14, 2024. Search terms included “Systemic lupus erythematosus,” “Air Pollutants,” and “Environmental Exposure.” The complete search strategy is detailed in **Supplementary Material Tables 1**, **2**.

### 2.2 Eligibility criteria

Studies were included based on the following criteria: (1) prospective or retrospective cohort designs or case-control studies; (2) investigations of the association between SLE and air pollution; (3) primary outcomes relating air pollution or environmental exposure to SLE risk, reported as hazard ratios (HR), odds ratios (OR), or relative risks (RR) during follow-up; (4) publications in English.

Exclusion criteria included: (1) duplicate studies; (2) reviews, conference abstracts, comments, or letters; (3) incomplete data or lack of outcomes of interest; (3)Time series study evaluating air pollution and the risk of hospitalization for SLE.

### 2.3 Study selection

Study selection was performed by two reviewers (YL Xu and HJ Pan), who independently screened literature according to the eligibility and exclusion criteria. If results were consistent, the final analysis proceeded; discrepancies were resolved by consulting full texts and discussing within the group.

### 2.4 Data extraction

Data extraction was conducted independently by the two reviewers (YL Xu and HJ Pan) using Microsoft Excel to create a data extraction table. This table captured information such as the first author, publication year, study type, sample size, follow-up duration, age, SLE diagnosis, and adjusted confounders. Extracted data were cross-checked, and discrepancies were resolved through group discussions.

### 2.5 Quality assessment

The Newcastle-Ottawa Scale (NOS) was employed to assess the quality of included studies. The NOS ranges from 0 to 9 points, with higher scores indicating better quality. It evaluates participant selection (4 points), comparability between groups (2 points), and exposure measurement (3 points). NOS scores of ≥7, 4–6, and 0–3 are classified as high, medium, and low quality, respectively.

### 2.6 Statistical analysis

The adjusted OR and 95% CI from each study were utilized to assess the association between air pollution, emerging chemical pollutants and SLE. We selected random or fixed effects models based on heterogeneity test results. Heterogeneity was considered high if P < 0.1 or I² > 50%. Due to the inherent heterogeneity in meta-analyses—clinical, methodological, and statistical—we employed a random effects model. To explore the sources of heterogeneity, we conducted *a priori* subgroup analyses based on predefined variables, including study design (cohort vs. case-control), age (adult vs. non-adult), population characteristics (Asian vs. non-Asian populations), and diagnostic criteria. These variables were selected based on clinical plausibility and prior evidence of their potential impact on the risk of SLE. For each subgroup, pooled risk ratios (RRs) with 95% confidence intervals (CIs) were calculated using the random-effects model. Heterogeneity within each subgroup was evaluated by the I^2^ statistic, and differences between subgroups were assessed by comparing the magnitudes of effect sizes and the overlap of confidence intervals. Sensitivity analyses were conducted to evaluate the robustness of results, and a case-by-case elimination method was used to explore sources of heterogeneity. Subgroup analyses were performed based on air pollution type, age, and study type. Publication bias was assessed using funnel plots and Egger’s regression tests.

## 3 Results

### 3.1 Literature search

**Figure 1:**
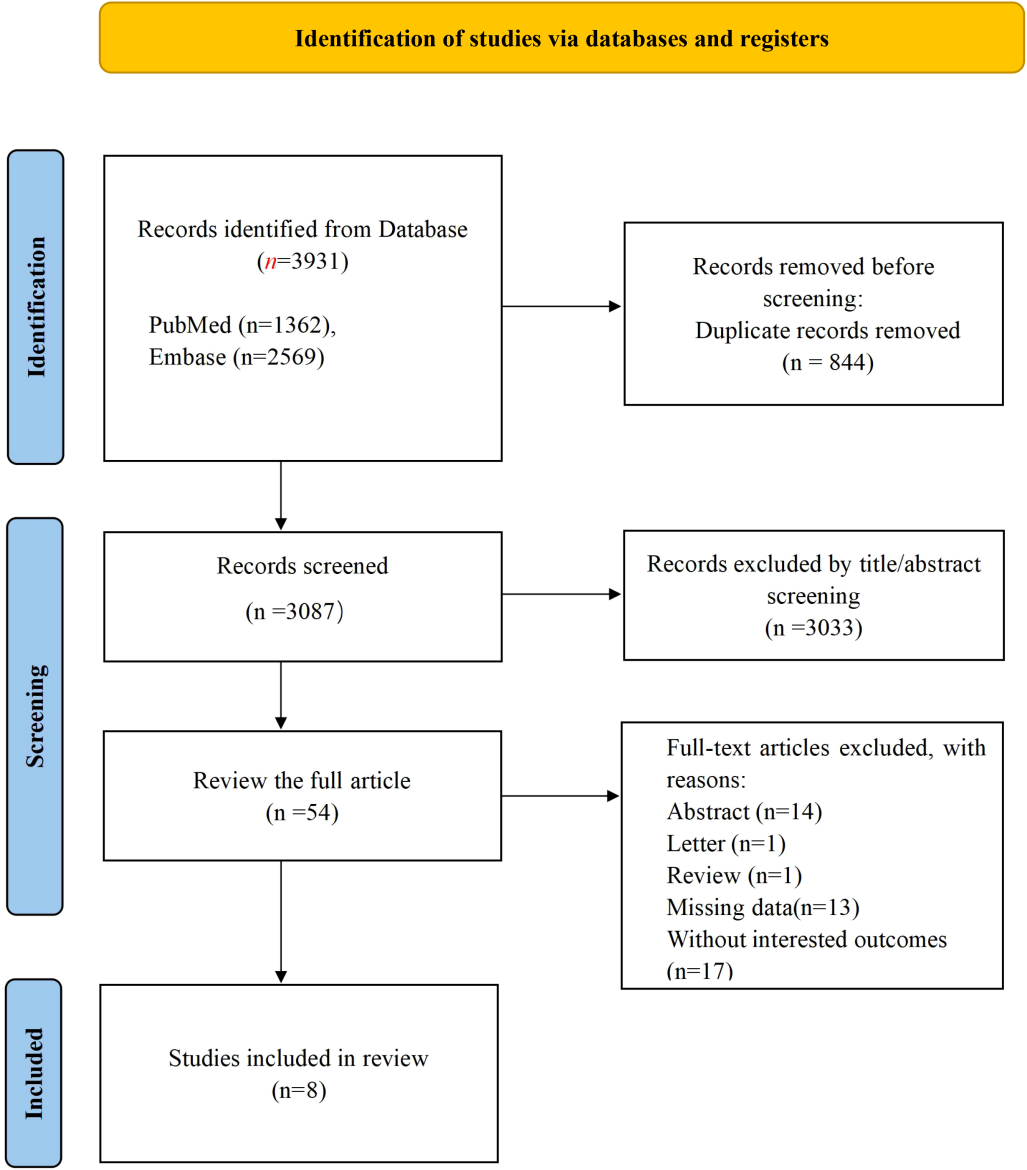
Literature screening.

The initial literature search identified 3,931 pertinent documents, comprising 1,362 articles from PubMed and 2,569 from EMBASE. These records were imported into Note Express reference management software. Upon screening titles and abstracts, irrelevant records were excluded, resulting in 54 articles for full-text evaluation. Ultimately, 8 studies met the eligibility criteria (21–28) and were included in the analysis, as depicted in **Figure 1**.

### 3.2 Basic characteristics

This meta-analysis encompasses 8 studies involving a total of 1,390,348 individuals, published between 2018 and 2024. Among these, four studies were retrospective cohort studies and four were case-control studies. The primary characteristics of the included subjects are outlined in **Table 1**. All eight included studies (21–28) reported associations between air pollution exposure and SLE. The diagnostic criteria for SLE varied across studies, with most using established classification systems (ACR or ICD criteria), while one study (26) did not explicitly report the diagnostic criteria used.

**Table 1:** Characteristics of studies included in the meta-analysis.

Author	Year	Country	Study type	Follow-up years*	Diagnostic criteria	Sample size	Age(Mean ± SD)	Confounders adjusted	Measures methods/Sample collection
Meiqi Xing	2024	UK	Cohort	2006-2020 (14 years)	(ICD-10) codes (M320, M321, M328, M329) and ICD-9 code 7100	459,815 patients included 459,416 patients without SLE and 399 patients newly diagnosed with SLE	56.6 ± 8.1	age, sex, ethnicity, household income, employment status, smoking status, drinking status, and BMI	A combined methodology of Land-Use Regression (LUR) model and satellite-based hybrid modeling
Guohua He	2024	China	Cohort	2001-2020 (19 years)	criteria of LN	342 patients diagnosed as cLN	<18	age, sex	Tracking Air Pollution in China (TAP) dataset
Yiyu Wang	2024	China	Case-control	NA	ACR criteria for SLE or the criteria of the International Collaborative Clinic for Systemic Lupus	168 female SLE patients and 175 controls	case group:38.2 ± 12	unadjusted	Collection of morning midstream urine samples from participants
							control group:39.6 ± 8.2		

Yan He	2023	China	Case-control	NA	SLICC classification criteria	100 SLE patients and 100 controls	>18	BMI、smoking, drinking, hypertension and leukocyte	Venous blood samples from participants
Jun Seok Park	2021	South Korea	Cohort	2002-2015 (13years)	M32 in ICD-10	In total, 230,034 participants and 40 participants were diagnosed with SLE	>=20	age (categorical: 20–34, 35–49, 50–64 and 65); sex (categorical: men and women); area of resi dence (categorical: Seoul, Busan and Incheon); and household income derived from the insurance premium(categorical: first, second, third and 4th quartiles)	Monitoring data from the Air Korea database
Kuo-Tung Tang	2019	China	Case-control	NA	NA	1858 SLE patients and 7432 controls	44.8 ± 16.6	age, sex, levels of urbanisation and family income	Air pollution monitoring data from Taiwan’s environmental protection agency and spatial interpolation methods

Chau-Ren Jung	2019	China	Cohort	2001-2010 (10 years)	ICD-9-CM code:7100	In total, 682,208 and 1,292 participants were diagnosed with SLE during the 2001–2010 follow-up period	43.26 ± 13.64	age, sex, SES, cerebrovascular disease, CKD,COPD, coronary artery disease, hyperlipidemia, and hypertension	A combined methodology of Land-Use Regression (LUR) model and satellite-based hybrid modeling
Paola G. Conde	2018	Brazil	Case-control	9 years	ACR criteria for SLE	30 SLE patients and 86 controls	0-9	unadjusted	Monitoring data from the Environmental Agency of the State of São Paulo, Brazil (CETESB)

cLN, Childhood-onset Lupus nephritis; ICD, International Classification of Diseases; BMI, body mass index; SLICC, systemic lupus international collaborative clinics; DOW, day of week; SES, socioeconomic status; CKD, chronic kidney disease; COPD, chronic obstructive pulmonary disease; ACR, American College of Rheumatology.

*Follow-up refers to the duration of follow-up time in cohort studies; case-control studies marked as NA.

### 3.3 Quality assessment

The average quality score across the studies was ≥ 6 points, comprising one studies scoring 6 points, three studies scoring 7 points, three studies scoring 8 points, and one study scoring 9 points. This distribution underscores the high quality of research incorporated in this meta-analysis. The NOS scoring scale is detailed in **Table 2**.

**Table 2:** Newcastle-Ottawa quality of cohort studies.

Study	Year	Selection	Comparability	Outcome	Total
Chau-Ren Jung	2019	★★	★	★★★	6
Jun Seok Park	2021	★★★★	★★	★★	8
Guohua He	2024	★★★	★	★★★	7
Meiqi Xing	2024	★★★★	★★	★★★	9
Paola G. Conde	2018	★★★★	★★	★★	8
Kuo-Tung Tang	2019	★★★	★★	★★	7
Yan He	2023	★★★	★★	★★	7
Yiyu Wang	2024	★★★★	★★	★★	8

### 3.4 Meta-analysis

#### 3.4.1 Air pollution and the association with SLE

**Figure 2:**
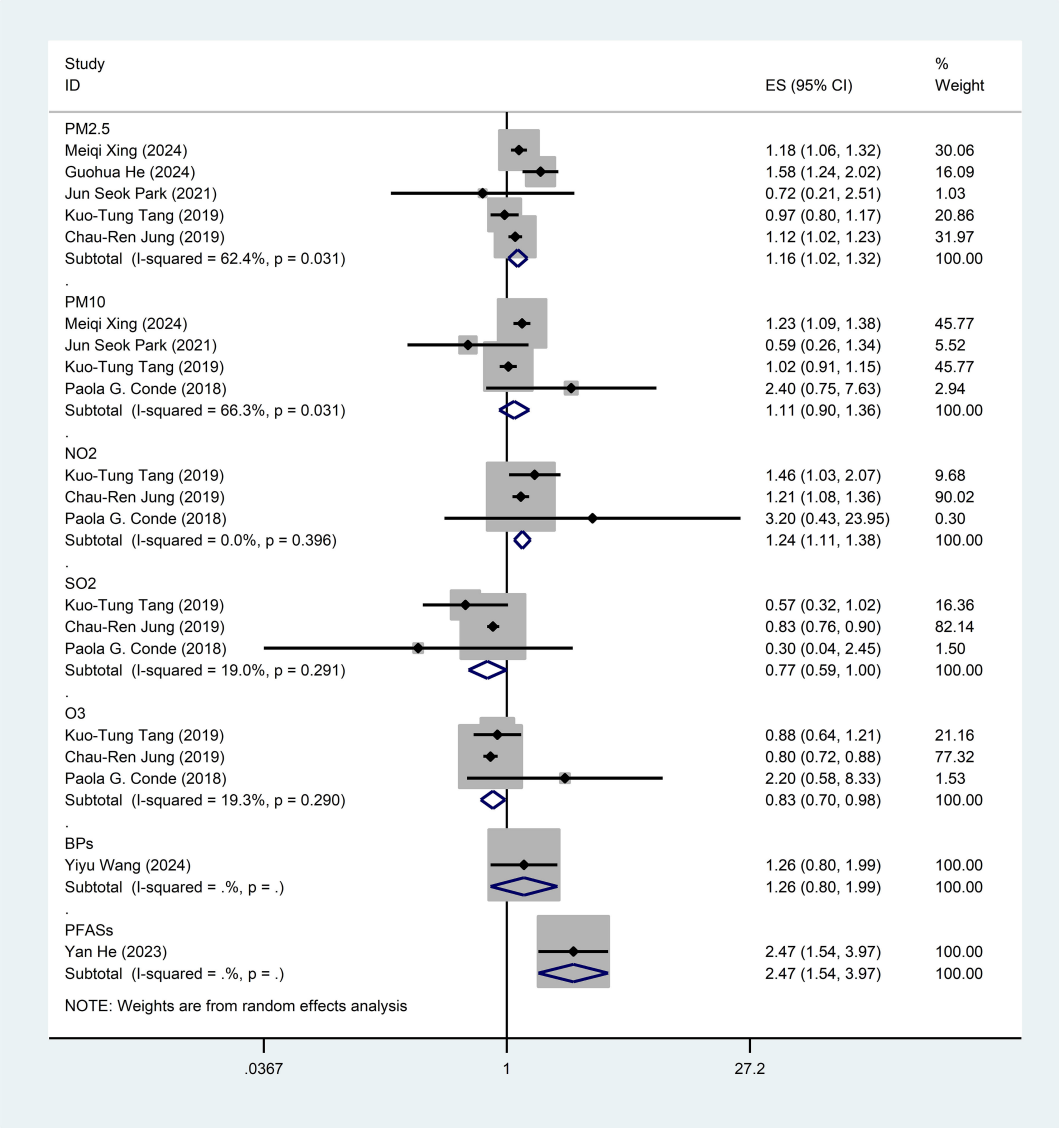
Forest plot for the associations between air pollution, emerging chemical pollutants and SLE. PM_2.5_, Particulate Matter 2.5; PM_10_, Particulate Matter 10; NO_2_, nitrogen dioxide; SO_2_, sulfur dioxide; O_3_, ozone; BPs, bisphenol analogue such as bisphenol A(BPA), bisphenol F(BPF), and bisphenol S(BPS); PFASs, Perfluoroalkyl and polyfluoroalkyl substances.

This meta-analysis included eight studies (21–28) investigating the association between air pollution exposure and SLE, including case-control studies and cohort studies. Five studies analyzed the association between PM_2.5_ and SLE [OR = 1.16, 95% CI (1.02-1.32), I^2^ = 62.4%, p=0.031, **Figure 2**], indicating a significant positive correlation. Four studies examined the effects of inhalable PM_10_ [OR = 1.11, 95% CI (0.90-1.36), I^2^ = 66.3%, p=0.031], showing a positive correlation trend but not reaching statistical significance. Three studies assessed the exposure effects of NO_2_ [OR = 1.24, 95% CI (1.11-1.38), I^2^ = 0.0%, p=0.603], indicating a significant positive correlation. Exposure to SO_2_ was not significantly associated with SLE [OR = 0.77, 95% CI (0.59-1.00), I^2^ = 19.0%, p=0.291]. Three studies assessed the effects of O_3_ [OR = 0.83, 95% CI (0.70-0.98), I^2^ = 19.3%, p=0.290], both showing a negative correlation but only O_3_ reaching statistical significance. One study examined the exposure effects of PFASs [OR = 2.47, 95% CI (1.54-2.57)]. One study analyzed the effects of BPs [OR = 1.26, 95%CI (0.80-1.99)]. Sensitivity analyses confirmed that none of the studies altered the overall effect, indicating the reliability of these findings regarding air pollution’s impact on SLE (**Supplementary Figure A**).

#### 3.4.2 Subgroup analysis

Based on pre-specified clinically relevant variables (age, study design, ethnicity, and diagnostic criteria), we conducted subgroup analyses to systematically assess potential effect modification of the association between air pollution and SLE (**Table 3**). Six studies (23–28) in adult populations showed a positive but non-statistically significant association [OR = 1.10, 95% CI (0.91–1.33), I^2^ = 87.4%, P < 0.001]. Meanwhile, two studies (21, 22) targeting non-adult populations showed a stronger and statistically significant association [OR = 1.58, 95% CI (1.25–1.99), I^2^ = 0.0%, P = 0.938]. However, the number of studies in the non-adult subgroup was limited (n = 2). So, although a larger effect size was observed, caution is warranted when interpreting this difference. Analysis by study design showed that cohort studies (22, 24, 25, 28) demonstrated a moderate positive association [OR = 1.12, 95% CI (0.88–1.42), I^2^ = 88.3%, P < 0.001], while case - control studies (21, 23, 26, 27) yielded higher but less stable estimates [OR = 1.40, 95% CI (0.85–2.29), I^2^ = 84.0%, P < 0.001]. In the ethnic subgroup analysis, six Asian studies yielded an OR of 1.17 [95% CI (0.92-1.48)] with high heterogeneity (I^2^ = 85.8%, P < 0.001), while two non-Asian studies showed an OR of 0.18 [95% CI (0.10-0.26)] with minimal heterogeneity (I^2^ = 0.0%, P = 0.537). When stratified by diagnostic criteria, two studies using ACR criteria reported an OR of 1.33 [95% CI (0.90-1.96)] with no heterogeneity (I^2^ = 0.0%, P = 0.672), whereas three studies using ICD criteria demonstrated an OR of 1.00 [95% CI (0.77-1.29)] but significant heterogeneity (I^2^ = 88.5%, P < 0.001). Significant heterogeneity exists between subgroups (I^2^ > 80% in most analyses), and there is an imbalance in the number of studies between adult and non - adult populations. This suggests that these stratified results should be interpreted with caution. Although the pattern suggests a possible age-dependent effect, the current evidence remains inconclusive due to limited pediatric population data and methodological differences in study designs.

**Table 3:** Subgroup analysis for the associations between air pollution, emerging chemical pollutants and SLE.

Subgroups	Included studies	OR (95% CI)	Heterogeneity	
			*I ^2^* (%)	*P* value
Age				
Adult	6	1.10 (0.91, 1.33)	87.4%	0.000
Non-adult	2	1.58 (1.25, 1.99)	0.0%	0.938
Study type				
Cohort study	4	1.12 (0.88, 1.42)	88.3%	0.363
Case-control studies	4	1.40 (0.85, 2.99)	84.0%	0.000
Ethnicity				
Asian	6	1.17 (0.92, 1.48)	85.8%	0.000
Non-Asian	2	0.18 (0.10, 0.26)	0.0%	0.537
Diagnose criteria				
ACR	2	1.33 (0.90, 1.96)	0.0%	0.672
ICD	3	1.00 (0.77, 1.29)	88.5%	0.000

ACR, American College of Rheumatology; ICD, International Classification of Diseases.

### 3.5 Publication bias

**Figure 3:**
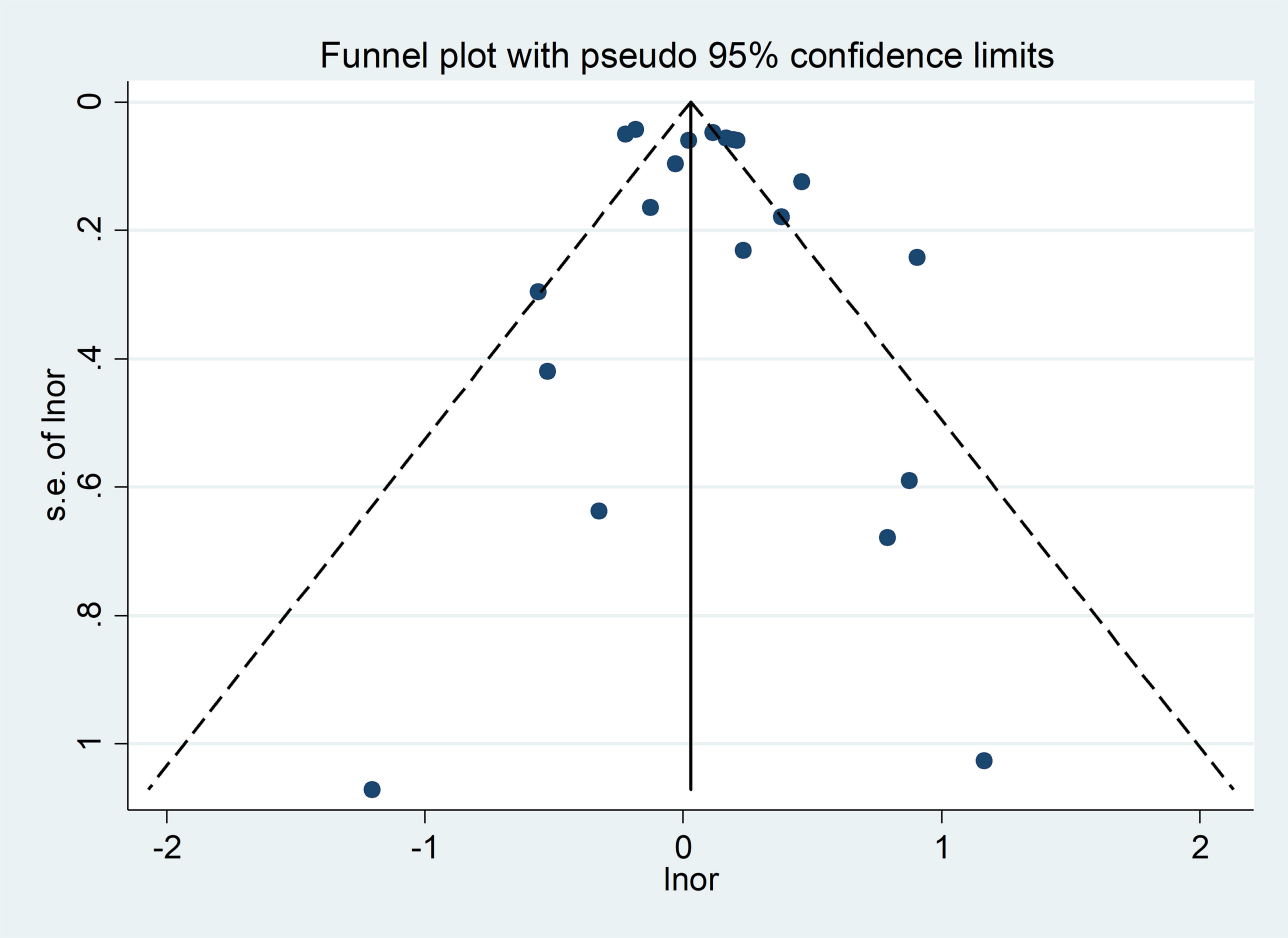
Funnel plot for air pollution and emerging chemical pollutants in SLE patients.

Visual inspection of the funnel plots revealed a rough symmetry, suggesting no significant publication bias in the association between SLE and air pollution (**Figure 3**). Consequently, we have a high degree of confidence in the validity of our findings. Furthermore, the Egger regression test yielded a p-value of 0.352 (p > 0.05), reinforcing the absence of publication bias in our meta-analysis.

### 3.6 Results of heterogeneity assessment

In this meta-analysis, the moderate heterogeneity observed for PM_2.5_ (I^2^ = 56.2%) and PM_10_ (I^2^ = 66.3%) may stem from multiple factors. In terms of population characteristics, the adult subgroup included studies from different regions and only partial studies controlled for socioeconomic status, while the non-adult subgroup showed 0.0% heterogeneity but was limited by small sample size. Regarding study design, case-control studies (I^2^ = 84.0%) and cohort studies (I^2^ = 88.3%) differed in exposure assessment methods, with the former susceptible to recall bias and the latter’s model estimation potentially ignoring individual activity differences. In terms of exposure windows, the follow-up duration of cohort studies varied widely, and short-term exposure might fail to capture the long latency effect of SLE. Diverse measurement methods for pollutants, such as satellite data, dataset extraction, and spatial interpolation for PM_2.5_, also contributed. Additionally, sensitivity analysis using leave-one-out method showed persistent high heterogeneity after excluding individual studies, indicating that heterogeneity was driven by multiple factors including exposure windows, measurement methods, and population differences rather than bias from a single study.

In addition, among the eight included studies, only one study was available for each emerging pollutant exposure, making it impossible to calculate heterogeneity indices for PFASs and BPs.

## 4 Discussion

### 4.1 Main findings

This meta-analysis included eight cohort studies comprising 1,390,348 participants to investigate the association between air pollution exposure and SLE. The analysis demonstrated significant positive associations between SLE incidence and exposure to PM_2.5_ [OR = 1.16, 95%CI (1.02-1.32)] as well as nitrogen dioxide (NO_2_) [OR = 1.24, 95%CI (1.11-1.38)]. While these findings provide epidemiological evidence supporting an association between specific air pollutants and SLE, the observational nature of the included studies limits causal inference, and residual confounding cannot be excluded.

### 4.2 Interpretation of findings

A prior meta-analysis (20) focused on the association of PM_2.5_ with an increased risk of SLE, highlighting its correlation with SLE Disease Activity Index (SLEDAI) scores. Notably, the time-dependence of PM_2.5_’s impact was observed, with no significant association on the third day of exposure but a positive correlation on the sixth day.

In our meta-analysis, encompassing eight articles and 1,390,348 participants, we investigate the association between PM_2.5_ and various air pollutants with SLE.Our findings further confirm the significant association between PM_2.5_ and SLE. Notably, we observed a pronounced association between NO_2_ exposure and SLE. Moreover, we expanded the analysis to include specific chemical environmental pollutants (such as BPs, PFASs), revealing their substantial contribution to SLE.

By incorporating updated research data, our analysis offers a comprehensive assessment of the association between air pollution exposure and SLE, encompassing a wide array of pollutants.

We conducted subgroup analyses based on age and study design. The results showed that the effect size in the non-adult group [OR = 1.58, 95% CI (1.25-1.99)] was higher than that in the adult group [OR = 1.10, 95% CI (0.91-1.33)], suggesting a possible age-related difference. However, it is important to note that the analysis of the non-adult group was based on only two studies with a total of 372 cases, which is insufficient in terms of sample size and number of studies, limiting the reliability of this result. This finding requires further validation in prospective pediatric cohort studies, particularly in conjunction with recent reports of a declining trend in the incidence of childhood SLE (29) for more in-depth exploration. Second, subgroup analysis showed that the association between air pollution and SLE was consistent in both cohort studies [OR = 1.12, 95% CI (0.88-1.42)] and case-control studies [OR = 1.40, 95% CI (0.85-2.29)], although neither reached statistical significance. The effect size observed in case-control studies was larger, a pattern consistent with previously reported methodological differences between observational study designs, suggesting that methodological characteristics should be considered when interpreting assessments of air pollution’s health effects across different study designs.

In our study, although the Egger regression did not suggest significant publication bias (P = 0.352>0.05), the funnel plot showed that small sample studies clustered at the high end of the effect size. This phenomenon implies that there may be a small study effect. Further observation revealed that some of the small-sample studies had relatively low quality scores. This may be related to study design flaws due to small sample sizes, thus increasing the likelihood of the existence of small-sample study effects. For example, 30 patients with childhood-onset systemic lupus erythematosus (cSLE) and 86 healthy controls were selected for the study by Conde, P.G. et al. (21). The sample size was relatively small. This makes the results of the study potentially not representative enough to accurately reflect the relationship between environmental factors and the onset of cSLE in the overall population, and makes it impossible to completely exclude the influence of possible publication bias and small-scale effects of the study. He, G. et al. (23) analyzed the relationship between PFASs and SLE. Their study, included 100 normal and 100 SLE patients, had a limited sample size and was susceptible to the confounding influence of small-scale study effects.

Furthermore, we analyzed the impact of potential confounders that may have been present in the studies. Nearly half of the studies showed that socioeconomic status was a common potential confounder. In general, areas with poorer economic conditions tend to have poorer environmental quality. Residents are more likely to be exposed to high concentrations of PM and have a relatively higher risk of developing SLE. Also, economic conditions may influence residents’ diet and healthcare, which may indirectly affect the onset of SLE. Of the 8 studies included in this review, 4 controlled for socioeconomic status, making the results relatively robust (24–26, 28). However, some studies (21) suggest that occupational exposures or air pollutants of the mother during pregnancy may affect the development of the foetal immune system. In turn, the development of the foetal immune system has been linked to the development of cSLE. This suggests that our genetic susceptibility and occupational exposures may be potential factors influencing the results of the study. However, very few original studies (21) have moderated the two variables of occupational exposure and genetic susceptibility, a factor that contributes to the uncertainty of the study results.

Among the studies we included, air pollution detection methods varied, including environmental monitoring and self-reported exposure. Differences in measurement methods may affect pooled effect estimates to some extent, thereby interfering with accurate judgements of the air pollution-disease relationship. For example, Jung, C. R. et al. (24) used a 1 - km resolution land use regression (LUR) model and a satellite estimation model to estimate air pollutant concentrations. This approach can better reflect the spatial distribution of air pollutants in a large region and effectively capture the differences in pollution in different areas within a city. However, this method may not accurately reflect the actual exposure of individuals due to their different indoor activity times and frequencies; He, G. et al. (23) extracted the residential addresses of patients in their study and obtained air pollutant data with the help of the China Tracking Air Pollution (TAP) dataset. This dataset integrates multiple factors to a certain extent and enables a more comprehensive assessment of air pollutant exposure. These data have been integrated with various factors to some extent, providing a more comprehensive assessment of air pollution exposure. However, the veracity of the data remains susceptible to the influence of various factors, including but not limited to model assumptions and data fusion methodologies. As a consequence, this may introduce biases into the estimates of the pooled effects; Conde, P. G. et al. (21) used a questionnaire to assess exposure. Participants were asked to provide information including the mother’s occupational exposures during pregnancy and the home surroundings. This method of self-reporting exposure is simple and easy to administer, but suffers from recall bias and inaccurate information. Participants may not be able to accurately recall past exposures or may have biased perceptions of certain exposures, leading to errors in exposure data. Therefore, future studies should incorporate multiple methods to improve exposure measures to more accurately assess the relationship between air pollution and SLE and to improve study reliability.

At present, the biological pathogenesis of air pollutants inducing or influencing the occurrence of SLE is not well understood. Several views on its mechanism are mainly related to oxidative stress and immune disorders, and epigenetic alterations (30–32). Air pollution can disrupt helper T cell (Th) homeostasis. It can also activate nuclear factor-κB, which then regulates Th1. Th1 binds to aryl hydrocarbon receptors to regulate Th17 and regulatory T cells. This process triggers the production of pro-inflammatory cytokines (32, 33). This concept is supported by a recent study by Dellaripa et al., which demonstrated that air pollutant exposure can induce specific immune perturbations that are characteristic of early autoimmunity (34). PM, as a representative substance of air pollution, can cause or amplify oxidative stress due to the presence of heavy metals, organic carbon and other complex elements on its surface (35). PM from air pollution, when inhaled, produces oxidants locally in the alveoli, triggering local chronic inflammation. The toxicity of PM depends on its size, shape, and composition, and the transition metals (e.g., Fe, V, Cr, etc.) present in it can produce oxidative stress through a Fenton-type reaction, which can have a negative effect on cells (36, 37). For example, silica particles are toxic to macrophages, inducing cell death and exposing intracellular self-antigens to immune cells (38), which in turn triggers an immune response (39). Inhalation of nanoparticles stimulates alveolar macrophages, triggering an acute systemic inflammatory response, and airway inflammation leads to an increased secretion of pro-inflammatory mediators such as interleukin-8 and granulocyte-macrophage colony-stimulating factor, as well as an influx of neutrophils (40–42). In addition, PM may act as an adjuvant (43), inducing an immune response against otherwise non-immunogenic antigens (44). All of these processes may interfere with normal immune regulation, leading to immune imbalances and increasing the risk of autoimmune diseases. Furthermore, epigenetic modifications play a crucial role in the pathogenesis of SLE (45). Abnormal methylation patterns can lead to aberrant gene activation. For example, hypomethylation of genomic DNA and immune - related genes in CD4+ T cells can cause overexpression of certain genes, thus triggering an autoimmune response (46, 47). Histone modifications, such as acetylation, phosphorylation, ubiquitination, and methylation, affect chromatin structure and gene expression (48). RNA methylation, particularly N6 - methyladenosine (m6A) modification (49), is also implicated in SLE. Downregulation of the demethylase AlkB homolog 5 (ALKBH5) in PBMCs and T cells of SLE patients inhibits apoptosis and promotes T - cell proliferation (50). Upregulation of methyltransferase 3 (METTL3) in the kidneys of SLE patients promotes IRF4 - mediated plasma cell infiltration, resulting in kidney damage (51). In summary, these epigenetic modifications interact with each other and affect the immune system, ultimately driving the occurrence and progression of SLE.

### 4.3 Implications and limitations

This study conducted a systematic review and meta-analysis to comprehensively assess the epidemiological association between air pollution exposure and the incidence of SLE. Through subgroup analysis, we further explored the specific association patterns between different types of air pollutants (such as PM_2.5_ and PM_10_) and SLE. These findings provide new epidemiological evidence for a deeper understanding of the potential role of environmental factors in the pathogenesis of SLE and offer guidance for future mechanistic studies.

Despite the significant contributions of this study, several limitations warrant consideration. Firstly, the restriction to PubMed/Embase searches might raise concerns about incomplete literature coverage. Although our meta-analysis searched only PubMed and Embase, this approach is methodologically justified. Embase complements PubMed by uniquely covering European and Asian journals in air pollution research (52), ensuring geographical diversity. Methodological analyses further show that including additional databases (e.g., Web of Science, Scopus, or Cochrane Library) identifies fewer than 3% more eligible studies in observational meta-analyses (53, 54). Given the high specificity of our topic—air pollution and SLE—this marginal increase is methodologically insignificant. Our study aligns with the AMSTAR 2 tool’s recommendation (55) to prioritize ‘reasonable’ database selection over exhaustive searches.

Second, Although SLE exhibits well-documented sex disparities in prevalence and clinical manifestations, our meta-analysis could not perform a robust sex-stratified analysis due to insufficient reporting of sex-specific associations between air pollution exposure and SLE in the included studies. Despite our initial intention to explore potential effect modification by sex, the original studies either lacked stratified outcome data or did not explicitly examine interactions between air pollution and sex, precluding meaningful subgroup comparisons. We hope that future research will incorporate more detailed data on sex-specific responses to air pollution, thereby enriching the understanding of how sex may modify the association between air pollution and SLE.

Third, the temporal relationship between pollution exposure and SLE onset exhibited substantial heterogeneity across studies, profoundly influencing the interpretability of exposure-response associations. For instance, studies adopting longitudinal cohort designs (24, 28) evaluated cumulative exposure over 10–15 years prior to SLE diagnosis, aligning with the disease’s postulated latency period (11.77 years median follow-up). Such long-term assessments captured PM_2.5_/NO_2_ concentrations during critical preclinical phases, identifying threshold effects (e.g., NO_2_ 28–38 ppb, PM_2.5_ 18–46 μg/m^3^) that correlated with increased risk. In contrast, cross-sectional or case-control studies (23, 27) measured biomarkers (PFASs/BPs) at the time of diagnosis without clear temporal linkage to disease initiation, precluding inference of exposure-onset causality. Studies focusing on pediatric SLE (22) further highlighted temporal complexity, with perinatal exposures (maternal occupational vapor/secondhand smoke) and childhood PM_2.5_ demonstrating associations with disease activity and renal failure. However, short-term evaluations (25, 26) using 2-4-year exposure windows (2008-2011) might have underestimated chronic risks, as seen in RA-specific analyses where PM_2.5_ exposure 3–5 years prior to diagnosis showed stronger associations (aHR=1.74) than concurrent measurements. This methodological variance introduces bias in pooled effect estimates. Shorter-term assessments (e.g., ≤2 years) in cross-sectional designs (26) may reflect disease-related behavioral changes rather than causal exposure, while self-reported data (21) risk recall bias. For SLE—a chronic disease with protracted preclinical phases—standardizing exposure windows to 5–10 years before diagnosis (28) in future meta-analyses will enhance causal inference, particularly when integrating biological plausibility.

It should also be noted that this meta-analysis has limitations in the geographic distribution of included studies: among all 8 included studies, 6 focused on Asian populations. This lack of diversity in study populations partially limits the generalizability of the findings to other ethnicities and regions (such as North America and Europe). It is important to note that the impact of environmental exposures on autoimmune diseases is mediated by multiple factors, including variations in genetic susceptibility, lifestyle characteristics, baseline disease prevalence, and the specific composition and concentration levels of pollutants in the environment. These factors often exhibit significant regional differences. Therefore, the pooled effect estimates derived from this study may not be directly generalizable to Western populations. This limitation reveals a critical gap in the current literature and the urgent need for large-scale, rigorously designed epidemiological studies in non-Asian populations to examine the links between air pollution, emerging chemical exposures, and SLE. Such studies are essential to validate the reliability of the findings from this research and to refine our understanding within a broader population context.

Additionally, while we identified significant associations for BPs and PFASs in individual studies, the lack of comparable data precluded their inclusion in quantitative synthesis. Future systematic reviews with expanded chemical exposure categories are warranted.

## 5 Conclusion

This meta-analysis revealed a significant association between the incidence of SLE and exposure to specific air pollutants, particularly PM_2.5_ and NO_2_. Exposure to emerging chemicals, such as PFASs, showed a strong association, but due to limited research, the association was not sufficiently robust. However, the relationship between BPs and SLE remains unclear, as existing evidence only suggests a non-significant positive trend. These findings emphasize the need for further large-scale epidemiological and mechanistic studies to clarify the role of environmental pollutants in the pathogenesis of SLE. Improving air quality and regulating known risk factors such as PM_2.5_ and NO_2_ may help reduce the risk of SLE, but further research is needed to validate the potential impacts of other environmental exposures.
