# Supplementary material for: Air pollution, emerging chemical exposures, and systemic lupus erythematosus: a meta-epidemiology study
Source: Front Immunol. 2025 Oct 17;16:1613441. doi: 10.3389/fimmu.2025.1613441 (PMC12575304; doi:10.3389/fimmu.2025.1613441)
Supplement: Supplementary file 1 [file Table1.docx]

Supplementary Material

**The retrieval strategies and retrieval results of each database are shown in Tables 1-2**.

Table 1: PubMed

| No. | Content | Result |
| --- | --- | --- |
| #1 | Search:(((((((("Air Pollutants"[Mesh]) OR "Air Pollution"[Mesh]) OR "Environmental Exposure"[Mesh]) OR "Vehicle Emissions"[Mesh]) OR "Particulate Matter"[Mesh]) OR "Nitrogen Oxides"[Mesh]) OR "Ozone"[Mesh]) OR "Sulfur Dioxide"[Mesh]) OR "Fossil Fuels"[Mesh] Sort by: Most Recent | 614,972 |
| #2 | Search: ((((((((((((((((((((((((((Air[Title/Abstract]) OR (ambient[Title/Abstract])) OR (atmospher*[Title/Abstract])) OR (Emission*[Title/Abstract])) OR (anthropogenic[Title/Abstract])) OR (particulate matter[Title/Abstract])) OR (ambient particulate[Title/Abstract])) OR (PM1[Title/Abstract])) OR (PM2[Title/Abstract])) OR (PM10*[Title/Abstract])) OR (ultrafine particulate*[Title/Abstract])) OR (ultrafine particle*[Title/Abstract])) OR (UFP[Title/Abstract])) OR (Coarse particle*[Title/Abstract])) OR (Soot[Title/Abstract])) OR (Black smoke[Title/Abstract])) OR (Black carbon[Title/Abstract])) OR (elemental carbon[Title/Abstract])) OR (wood smoke[Title/Abstract])) OR (power generation[Title/Abstract])) OR (indust*[Title/Abstract])) OR (combustion[Title/Abstract])) OR (smelting[Title/Abstract])) OR (construction[Title/Abstract])) OR (demolition[Title/Abstract])) OR (burning[Title/Abstract])) OR (residential[Title/Abstract]) Sort by: Most Recent | 1,423,000 |
| #3 | Search: ((((((((("Air Pollutants"[Mesh]) OR "Air Pollution"[Mesh]) OR "Environmental Exposure"[Mesh]) OR "Vehicle Emissions"[Mesh]) OR "Particulate Matter"[Mesh]) OR "Nitrogen Oxides"[Mesh]) OR "Ozone"[Mesh]) OR "Sulfur Dioxide"[Mesh]) OR "Fossil Fuels"[Mesh]) OR (((((((((((((((((((((((((((Air[Title/Abstract]) OR (ambient[Title/Abstract])) OR (atmospher*[Title/Abstract])) OR (Emission*[Title/Abstract])) OR (anthropogenic[Title/Abstract])) OR (particulate matter[Title/Abstract])) OR (ambient particulate[Title/Abstract])) OR (PM1[Title/Abstract])) OR (PM2[Title/Abstract])) OR (PM10*[Title/Abstract])) OR (ultrafine particulate*[Title/Abstract])) OR (ultrafine particle*[Title/Abstract])) OR (UFP[Title/Abstract])) OR (Coarse particle*[Title/Abstract])) OR (Soot[Title/Abstract])) OR (Black smoke[Title/Abstract])) OR (Black carbon[Title/Abstract])) OR (elemental carbon[Title/Abstract])) OR (wood smoke[Title/Abstract])) OR (power generation[Title/Abstract])) OR (indust*[Title/Abstract])) OR (combustion[Title/Abstract])) OR (smelting[Title/Abstract])) OR (construction[Title/Abstract])) OR (demolition[Title/Abstract])) OR (burning[Title/Abstract])) OR (residential[Title/Abstract])) Sort by: Most Recent | 1,860,018 |
| #4 | Search:"Lupus Erythematosus, Systemic"[Mesh] Sort by: Most Recent | 69,303 |
| #5 | Search:(((systemic lupus erythematosus[Title/Abstract]) OR (Lupus[Title/Abstract])) OR (SLE[Title/Abstract])) OR (lupus erythematosus[Title/Abstract]) Sort by: Most Recent | 99,739 |
| #6 | Search: ("Lupus Erythematosus, Systemic"[Mesh]) OR ((((systemic lupus erythematosus[Title/Abstract]) OR (Lupus[Title/Abstract])) OR (SLE[Title/Abstract])) OR (lupus erythematosus[Title/Abstract])) Sort by: Most Recent | 108,219 |
| #7 | Search: (((((((((("Air Pollutants"[Mesh]) OR "Air Pollution"[Mesh]) OR "Environmental Exposure"[Mesh]) OR "Vehicle Emissions"[Mesh]) OR "Particulate Matter"[Mesh]) OR "Nitrogen Oxides"[Mesh]) OR "Ozone"[Mesh]) OR "Sulfur Dioxide"[Mesh]) OR "Fossil Fuels"[Mesh]) OR ((((((((((((((((((((((((((((Air pollut*[Title/Abstract]) OR (Air pollut*[Title/Abstract])) OR (ambient[Title/Abstract])) OR (atmospher*[Title/Abstract])) OR (Emission*[Title/Abstract])) OR (anthropogenic[Title/Abstract])) OR (particulate matter[Title/Abstract])) OR (ambient particulate[Title/Abstract])) OR (PM1[Title/Abstract])) OR (PM2[Title/Abstract])) OR (PM10*[Title/Abstract])) OR (ultrafine particulate*[Title/Abstract])) OR (ultrafine particle*[Title/Abstract])) OR (UFP[Title/Abstract])) OR (Coarse particle*[Title/Abstract])) OR (Soot[Title/Abstract])) OR (Black smoke[Title/Abstract])) OR (Black carbon[Title/Abstract])) OR (elemental carbon[Title/Abstract])) OR (wood smoke[Title/Abstract])) OR (power generation[Title/Abstract])) OR (indust*[Title/Abstract])) OR (combustion[Title/Abstract])) OR (smelting[Title/Abstract])) OR (construction[Title/Abstract])) OR (demolition[Title/Abstract])) OR (burning[Title/Abstract])) OR (residential[Title/Abstract]))) AND (("Lupus Erythematosus, Systemic"[Mesh]) OR ((((systemic lupus erythematosus[Title/Abstract]) OR (Lupus[Title/Abstract])) OR (SLE[Title/Abstract])) OR (lupus erythematosus[Title/Abstract]))) Sort by: Most Recent | 1362 |

Table 2 Embase

| No. | Content | Result |
| --- | --- | --- |
| #1 | 'air pollutant'/exp OR 'air pollution'/exp OR 'environmental exposure'/exp OR 'exhaust gas'/exp OR 'particulate matter'/exp OR 'nitrogen oxide'/exp OR 'ozone'/exp OR 'sulfur dioxide'/exp OR 'fossil fuel'/exp | 394,945 |
| #2 | 'air'/exp OR air OR ambient:ab,ti OR atmospher*:ab,ti OR emission*:ab,ti OR anthropogenic:ab,ti OR 'particulate matter':ab,ti OR 'ambient particulate':ab,ti OR pm1:ab,ti OR pm2:ab,ti OR pm10*:ab,ti OR 'ultrafine particulate*':ab,ti OR 'ultrafine particle*':ab,ti OR ufp:ab,ti OR 'coarse particle':ab,ti OR soot:ab,ti OR 'black smoke':ab,ti OR 'black carbon':ab,ti OR 'elemental carbon':ab,ti OR 'wood smoke':ab,ti OR 'power generation':ab,ti OR indust*:ab,ti OR combustion:ab,ti OR smelting:ab,ti OR construction:ab,ti OR demolition:ab,ti OR burning:ab,ti OR residential:ab,ti | 1,771,072 |
| #3 | #1 OR #2 | 1,992,763 |
| #4 | 'systemic lupus erythematosus'/exp | 137,823 |
| #5 | 'systemic lupus erythematosus'/exp OR 'systemic lupus erythematosus' OR (systemic AND lupus AND erythematosus) OR lupus:ab,ti OR sle:ab,ti OR 'lupus erythematosus':ab,ti | 179,235 |
| #6 | #4 OR #5 | 179,235 |
| #7 | #3 AND #6 | 2569 |

**SUPPLEMENTARY FIGURE A | Sensitivity analysis** **of the risk of air pollution in patients with SLE.**
